# Beyond Standard Half‐Life: Real‐world Pharmacokinetics of Efanesoctocog Alfa in a Single Centre

**DOI:** 10.1111/hae.70306

**Published:** 2026-05-07

**Authors:** Laurent Sattler, Agathe Herb, Léa Pierre, Jordan Wimmer, Manon Dolt, Olivier Feugeas, Dominique Desprez

**Affiliations:** ^1^ Laboratoire D'hématologie, Unité Hémostase Hôpitaux Universitaires de Strasbourg Strasbourg France; ^2^ Centre de Ressource et Compétences des Maladies Hémorragiques Rares Hôpitaux Universitaires de Strasbourg Strasbourg France

**Keywords:** efanesoctocog alfa, haemophilia A, pharmacokinetics, prophylaxis, real‐world study

## Abstract

**Introduction:**

Efanesoctocog alfa (EFA) is an ultra–extended half‐life factor VIII (FVIII) developed to address limitations of conventional prophylaxis in haemophilia A. Although the XTEND trials reported low interindividual pharmacokinetic (PK) variability, real‐world data remain important to better characterize PK profiles across patient subgroups.

**Aim:**

To report real‐world PK data for EFA.

**Methods:**

In this single‐centre study, adults and children (>6 years) with severe haemophilia A at Strasbourg University Hospital switched from a prior FVIII product to EFA. A simplified PK assessment was performed once or twice per patient after a 50 IU/kg infusion. FVIII activity was measured pre‐infusion and at 3, 24, 96 h, and 7 days post‐infusion. Patients were reassessed at steady state after ≥6 weeks.

**Results:**

In adults, mean (range) half‐life was 52 h (35–62). Mean incremental recovery at 3 h was 2.98 IU/dL per IU/kg (1.85–3.82). Mean FVIII trough levels were 17 IU/dL (5–27) at switch and 19 IU/dL (10–27) at steady state. Significant interindividual variability was observed. Half‐life correlated positively with VWF:Ag levels (*r* = 0.63; *p* < 0.05) and body weight (*r* = 0.62; *p* < 0.05). In children, mean half‐life was 53 h (48–59), mean recovery 2.05 IU/dL per IU/kg (1.86–2.26), and trough levels 13–14 IU/dL.

**Conclusions:**

PK profiles matched or exceeded those reported in the XTEND studies, with a slightly longer half‐life and higher trough FVIII levels. Notable interindividual variability highlights the value of PK assessments, even for ultra‐extended half‐life FVIII, to optimize individualized patient management.

## 1 Introduction

Haemophilia A is an inherited bleeding disorder caused by a deficiency in factor VIII (FVIII), leading to spontaneous or trauma‐related bleeding episodes and chronic joint morbidity that may progress to disabling arthropathy [1, 2]. Although current prophylactic treatments, whether replacement or non‐replacement therapies, have substantially improved patient management and prognosis, several limitations remain [3, 4].

The relatively short half‐life of conventional FVIII concentrates—both standard half‐life (SHL) and extended half‐life (EHL)—along with interindividual variability in plasma FVIII levels, and the need for frequent infusions represent major challenges in achieving continuous and individualized haemostatic protection with conventional FVIII therapy. These constraints may negatively affect treatment adherence, quality of life, and optimal bleeding control, thereby promoting the progression of haemophilic arthropathy [5, 6, 7].

Non‐replacement therapies provide convenient and effective prophylaxis; however, they offer limited flexibility in acute bleeding situations, and their contribution to joint health preservation remains indirect, relying on overall bleeding control [3, 8, 9].

In this context, efanesoctocog alfa (EFA; Altuvoct or Altuviiio, Sanofi–Sobi), a recombinant FVIII consisting of a B‐domain‐deleted, Fc fusion FVIII linked to the D'D3 domain of von Willebrand factor (VWF), and two XTEN polypeptides, represents a major therapeutic advance. It addresses an unmet medical need in haemophilia A prophylaxis by providing a marked extension of FVIII plasma half‐life (38–47 h, three‐ to fourfold longer than conventional FVIII concentrates) and sustained activity levels throughout the dosing interval [10].

Phase III clinical trials from the XTEND program, including XTEND‐1 in adolescents and adults and XTEND‐Kids in children under 12 years of age, demonstrated high haemostatic efficacy associated with low interindividual PK variability [11, 12]. Nevertheless, even with an overall stable and predictable profile, precise characterization of EFA PK across different patient subgroups (particularly according to age and body weight) remains essential. This approach aims to consolidate findings from pivotal studies, confirm key PK parameters (half‐life, peak levels, incremental recovery, exposure, clearance, and trough levels) and provide practical data to optimize individualized prophylaxis.

Laboratory assessment of FVIII activity under EFA treatment raises specific analytical challenges related to its molecular structure and unique mechanism of action. Initial evaluations using one‐stage assays (OSA) and chromogenic substrate assays (CSA) have shown that results may vary significantly depending on the reagent used. In the absence of a mimetic agent (i.e., emicizumab), preliminary recommendations suggest the use of standard‐calibrated OSA with selected reagents, such as Actin FSL, Synthafax, or CK Prest, which should be locally validated [13, 14, 15, 16].

Hence, this study presents real‐world PK data from a single centre, using a locally validated OSA.

## 2 Materials and Methods

### 2.1 Population Selection and Inclusion Criteria

In this single centre cohort, approved by the institution's ethics board (CE‐2025‐90), outpatients with severe haemophilia A (PwHA) at Strasbourg's Haemophilia Treatment Centre (HTC) who had previously been treated with SHL or EHL FVIII and had undergone a therapeutic switch to EFA were included.

Given their potential impact on PK and their collection in the pivotal studies [11, 12], the following variables were recorded: age, sex, weight, height, body mass index (BMI), geographic origin, blood group, von Willebrand factor antigen (VWF:Ag), history of FVIII inhibitors, HCV status (serology and viral load), and renal and hepatic function. HIV status (serology and viral load) and CD4 lymphocyte count were also collected, in light of the discontinuation of EFA due to a decrease in CD4 count in one patient in the XTEND study [12]. Finally, comorbidities were recorded for descriptive purposes to further characterize the cohort.

### 2.2 Switch Protocol and Pharmacokinetic (PK) Study

Patients transitioned from their previous treatment to EFA based on a shared decision between the HTC physician and the patient during a medical consultation. For each patient, after a 48‐hour washout of their usual anti‐haemophilic therapy, a simplified PK study was conducted following a single infusion of 50 IU/kg of EFA.

FVIII levels were measured at the following time points: pre‐infusion (H0), 3 h (H3), 24 h (H24), 96 h (H96), and 7 days (D7) post‐infusion. Patients were re‐evaluated at least 6 weeks after the switch, prior to the next injection, to assess the trough level at steady state (H0’).

In patients with suitable venous access, a second PK study at steady state (≥6 exposure days—EDs—corresponding to ≥6 weeks of prophylaxis) was performed. In addition to the pre‐injection (trough) level at steady state (H0’), FVIII levels were measured at 96 h (H96’) and 7 days (D7’) after infusion.

Anti‐FVIII antibody screening was performed at H0 and H0’. VWF: Ag was measured at H0.

### 2.3 Blood Sampling and Platelet‐Poor Plasma Preparation

Blood samples for all assays were collected in Vacuette tubes (Greiner Bio‐One) containing 0.109 M sodium citrate. Samples were spinned twice and stored at −80°C in polypropylene aliquots. Prior to analysis, samples were thawed by immersion in a 37°C water bath for 4 min.

### 2.4 Assays

FVIII activity was measured using a OSA with CK‐Prest and Immunodeficient VIII, calibrated with Unicalibrator, on a STA‐R Max (whole Diagnostica Stago, Asnières‐sur‐Seine, France). The assay was locally validated using plasma samples artificially spiked with EFA and further verified through participation in external quality assurance exercises. A local overestimation of approximately 30% was observed for EFA levels ≤20 IU/dL, decreasing to <20% for levels ≥50 IU/dL (data not shown).

FVIII inhibitor detection was performed using a Bethesda assay. First, samples were heated for 90 min at 56°C [17, 18], mixed with Cryocheck normal pooled plasma (Cryopep, Montpellier, France), and then incubated in a water‐bath at 37°C for 2 h. FVIII residual activity was then assessed using the aforementioned FVIII assay.

VWF:Ag was measured using Liatest VWF:Ag (Diagnostica Stago) on a STA‐R Max.

### 2.5 PK Parameter Calculation

Incremental recovery was expressed as FVIII activity per IU/kg, calculated based on the administered dose, patient body weight, and FVIII level at H3 (i.e., peak). Half‐life was estimated using the WAPPS‐Hemo software (Web‐Accessible Population Pharmacokinetic Service‐Haemophilia, McMaster University) [19].

### 2.6 Statistical Analyses

Statistical tests were performed using Prism v6.05 (GraphPad Software). Half‐life and trough levels after the first infusion and at steady state (after ≥6 EDs) were compared using Student paired test. Correlations were assessed using a non‐parametric Spearman test, between recovery rate and weight, and between EFA's half‐life and weight, age and VWF:Ag.

FVIII <1 IU/dL was considered as equal to 1 IU/dL for statistical analysis.

## 3 Results

### 3.1 Adult Population

The adult cohort consisted of 20 male PwHA included between March 2024 and December 2025, who were followed up from 6 weeks to 22 months. The mean (range) number of EDs was 36 (6–106).

The mean (range) age of participants was 44 years (18–71), with 11 patients above 50 years old. The mean (range) BMI was 29 kg/m^2^ (19–56): 3 patients were overweighed (25< BMI < 30), 4 had moderate obesity (30< BMI < 35), 1 had severe obesity (35< BMI < 40), and 3 had morbid obesity (BMI > 40).

All patients had normal hepatic and renal function at inclusion, defined respectively by AST/ALT values <1.5× the upper limit of normal and an estimated glomerular filtration rate (eGFR) >90 mL/min/1.73 m^2^.

Two patients had arterial hypertension and dyslipidemia, and only one reported a history of cardiovascular disease (atrial fibrillation). Comorbidities were overall stable and well controlled, with no known interactions with the treatment. Three patients reported regular but moderate alcohol consumption, and only one patient was an active smoker (12 pack‐years).

Three participants had chronic HIV infection, all receiving effective antiretroviral therapy, and 11 patients had a history of hepatitis C virus (HCV) infection.

Only one patient, aged 19 years at the time of switching, had a history of inhibitor development, with a historical peak of 7 BU/mL, which occurred at the age of 2 years; the inhibitor has remained negative for the past 16 years.

Detailed patient characteristics are presented in Table 1.

**TABLE 1:** Baseline demographic characteristics of the cohort.

Adult patient data (*N* = 20)	Value
Age, years, mean (range)	44 (18–71)
Weight, kg, mean (range)	85 (55–160)
Body mass index (BMI), kg/m^2^, mean (range)	29 (19–56)
Geographic origin, *n* (%)	
European ancestry	19 (95)
African ancestry	1 (5)
VWF:Ag, IU/dL, mean (range)	131 (85–242)
Blood group, *n* (%)	
Non‐O	10 (50)
O	10 (50)
HIV‐positive patients, *n*	3
Detectable HIV viral load, *n* (%)	0
CD4 count, cells/mm^3^, median (IQR)	726 (622–848)
HCV‐positive patients, *n* (%)	11
Detectable HCV viral load, *n* (%)	0

After administration of a mean (range) dose of 50 IU/kg (44–56) of EFA, the mean (range) FVIII level on D7 was 17 IU/dL (5–27). Four patients had trough levels below 15 IU/dL. The mean (range) half‐life was estimated at 52 h (35–62). The mean (range) recovery at H3 was 2.98 IU/dL per IU/kg (1.85–3.82). At H96, FVIII was above 40 IU/dL in 88% of patients. The mean (range) trough level at steady state (H0’) was 19 IU/dL (10–27), significantly higher than at D7 (*p* < 0.05) (Table 2). Inhibitor testing remained negative in the entire cohort during the entire follow‐up.

**TABLE 2:** PK data in the overall adult population.

	H0	H3	H24	H96	D7	H0’	Half‐life
Mean FVIII IU/dL (range) *N* = 20	6 (1–21)	156 (104–211)	133 (78–184)	54 (34–76)	17 (5–27)	19 (10–27)	52 h (35–62)

Ten patients underwent a second PK assessment at steady state (≥6 EDs), with a mean (range) half‐life of 53 h (44–64), significantly higher than after the first infusion (50 h; *p* < 0.05). After 7 days, the mean (range) trough FVIII level on D7′ was 21 IU/dL (13–27), once again significantly higher than after the first infusion (17 IU/dL, *p* < 0.05) (Table 3).

**TABLE 3:** Comparative PK data for 10 patients included in both PK studies.

First PKs	H0	H96	D7	Half‐life
Mean FVIII IU/dL (range) *N* = 10	6 (<1–21)	53 (34–68)	17 (5–29)	50 h (35–62)
Second PKs	H0’	H96’	D7’	Half‐life
Mean FVIII IU/dL (range) *N* = 10	20 (10–29)	62 (31–83)	21 (13–27)	53 h (44–64)

There was a significant correlation between EFA half‐life and VWF:Ag (*r* = 0.63; *p* < 0.05) as well as patient weight (*r* = 0.62; *p* < 0.05). Similarly, a statistically significant correlation was observed between recovery and weight (*r* = 0.68; *p* < 0.05).

### 3.2 Pediatric Population

The pediatric cohort included 3 boys aged 6, 9, and 13 years, without comorbidities. The mean (range) number of EDs was 10 (6–13). The mean (range) age was 9 years (6–13), with a mean (range) weight of 32 kg (22–47) and a mean (range) BMI of 18 kg/m^2^ (14–22). All children had normal hepatic and renal function, defined by AST/ALT values <1.5× the upper limit of normal and an estimated glomerular filtration rate (eGFR) >90 mL/min/1.73 m^2^.

No history of FVIII inhibitor was reported.

The mean (range) recovery was 2.05 IU/dL per IU/kg (1.86–2.26). On D7, the mean (range) FVIII trough level was 13 IU/dL (10–17). The mean (range) half‐life was 53 h (48–59). All three children were reassessed after ≥6 EDs, with a mean (range) FVIII trough level of 14 IU/dL (8–17).

## 4 Discussion

EFA is a novel recombinant FVIII engineered to substantially extend plasma half‐life while maintaining consistently high haemostatic protection. Unlike conventional FVIII products, its unique structure enables independence from VWF, representing a significant pharmacological and PK advancement [20, 21].

In our study on 20 adults PwHA, EFA demonstrated a mean half‐life of 52 h after a single infusion, and 53 h at steady state – higher than the 47 h reported in published literature [12]. Such difference may be related to the individual characteristics of our population (age, weight, well‐controlled comorbidities). This is a significant improvement for patients, as it is significantly higher than that any currently available FVIII products [22]. We found a mean incremental recovery of 2.98 IU/dL per IU/kg, consisting with previous data (3.00 IU/dL per IU/kg, although peak levels vary according to individual profiles), enabling high plasma concentrations after administration [12]. However, striking interindividual differences were noted in our cohort too, highlighting the relevance of individual PK study.

At H96, 88% patients maintained FVIII levels above 40 IU/dL, providing effective protection against bleeding episodes. Last, trough levels at D7 were consistently elevated, with a mean at 17 IU/dL after a single infusion (min: 5 IU/dL), and 19 IU/dL at steady state (min: 10 IU/dL), higher than that of XTEND who found a trough level of 15 IU/dL [12].

As reported in the literature, EFA's favorable PK profile and its ability to maintain high sustained FVIII activity levels is further supported by its low clearance (approximately 0.5 mL/h/kg), and a volume of distribution largely confined to the intravascular space (approximately 31 mL/kg) [23].

In the XTEND‐Kids study, which included children younger than 12 years, the half‐life was reported to be slightly shorter than in the XTEND study, which included patients aged 12 years and older (38, 42, and 47 h in children <6 years, children aged 6–12 years, and patients ≥12 years, respectively), while remaining markedly longer than that observed with conventional FVIII products. In our very small cohort, which included only two children younger than 12 years (and one aged 13 years), the estimated half‐life was 53 h (48–59), approaching values reported in adults. The incremental recovery at H3 was estimated at 2.05 IU/dL per IU/kg (1.77–2.26), and the mean trough level was 13 IU/dL. In the XTEND‐Kids study, weekly prophylactic infusions maintained FVIII levels above 40 IU/dL for approximately three days, after which levels gradually declined but remained above 10 IU/dL up to D7, ensuring haemostatic protection throughout the dosing interval. Overall, our observations appear broadly consistent with these previously reported data. However, the very small number of paediatric patients in our study precludes any firm conclusions, and these findings should therefore be interpreted with caution and confirmed in larger paediatric cohorts [11, 12].

Of note, interassay variability could contribute to our slightly higher half‐life and trough levels in both adult and pediatric population: while Actin FSL was used to assess FVIII in pivotal studies, it has been shown that the CK‐Prest, which was used in this study, tends to overestimate low EFA concentrations [14, 15, 16]. We indeed observed a local overestimation of approximately 30% for EFA levels ≤20 IU/dL, whereas this overestimation gradually decreased and remained below 20% for levels ≥50 IU/dL (data not shown).

These results indicate that EFA maintains protective plasma levels throughout the week, confirming its potential for effective once‐weekly prophylaxis. The increase observed in the second PK assessment (≥6 exposure days) may reflect accumulation without excessive exposure, which could theoretically increase thrombotic risk.

Although data from the XTEND‐1 study demonstrate relatively homogeneous PK profiles, performing individual PK assessments with EFA remains relevant and useful for personalized management, as is already standard practice for all previous FVIII concentrates [24]. Typically, several predictors of half‐life have been identified in conventional FVIII concentrates, including BMI, baseline VWF:Ag, blood group (O vs. non‐O), history of inhibitors, HCV infection, and hematocrit [25, 26, 27, 28]. We found a significant correlation between weight and EFA's half‐life and recovery. Moreover, we observed an unexpected correlation between half‐life and patients’ VWF:Ag levels (*p* < 0.05).

In a one‐compartment population PK model derived from patients enrolled in phase 1/2a and phase 3 studies, peak and trough FVIII levels, as well as the duration during which FVIII activity remained ≥40 IU/dL post‐dose, were positively correlated with body weight. In contrast, no association was found with other variables, including VWF:Ag levels, HCV or HIV status, and blood group. These findings are consistent with the expected VWF‐independent elimination of EFA, as the incorporation of the D'D3 domain prevents binding to endogenous VWF [29]. However, a real‐world study involving 54 PwHA reported that, in addition to body weight (in line with previous observations), baseline VWF activity is a significant predictor of trough FVIII levels [30]. Given the molecular structure of EFA, the observed association between half‐life, trough levels, and VWF:Ag may reflect the influence of underlying physiological factors rather than a direct dependence on circulating VWF.

These preliminary real‐world findings regarding the PK determinants of EFA remain incompletely understood and may be influenced by characteristics of the study population, which is often small and geographically limited, as well as by the assays used to measure FVIII activity. Consequently, further studies are needed to confirm or refute these observations and to better elucidate the underlying mechanisms.

Nevertheless, these observations confirm that the PK benefits seen in clinical trials are reproducible in real‐world practice. Another important point to highlight is the interindividual variability of PK parameters observed in routine care, further supporting the need for individualized monitoring of patients receiving EFA prophylaxis, as the observed range of values is very wide.

As in the XTEND‐1 and XTEND‐Kids studies, no development of FVIII inhibitors was observed in the included patients. EFA's complex structure reduces exposure of FVIII epitopes and alters antigen presentation, which may limit T‐cell activation. XTEN polypeptides are non‐immunogenic and increase the size of the complex without adding epitopes. The Fc fragment may also promote immune tolerance through regulatory mechanisms [31]. Consequently, the immunogenicity of the molecule is reduced, although the risk of inhibitor development remains to be confirmed in previously untreated patients (PUPs). However, cases of FVIII inhibitor development have been reported during EFA therapy, highlighting the need for careful monitoring [32].

This study presents several limitations: first, the small cohort size, particularly in the pediatric population. Second, FVIII was assessed with CK‐Prest, which may lead to an overestimation of FVIII at trough levels. Last, although trough levels and half‐life are favorable, the long‐term clinical impact on bleeding frequency and joint health will need to be evaluated in extended and comparative studies.

Data from our cohort show that EFA reproduces, and in some parameters slightly exceeds, the PK profiles observed in the XTEND studies, providing an extended half‐life, high incremental recovery, and sustained trough levels at D7. This validates its ability to provide effective once‐weekly prophylaxis in both adult and pediatric patients with haemophilia A.

## 5 Conclusion

In conclusion, this real‐world study on the switch of 23 patients with severe haemophilia A to EFA demonstrated highly satisfactory results, both biologically and clinically.

Monitoring FVIII levels under EFA requires close collaboration between clinicians and laboratory specialists, as a standardization of EFA monitoring is still required. Knowledge of potential mimetic agents, the use of validated reagents and appropriate methodologies, and contextual interpretation of results are essential to ensure reliable and safe monitoring in treated patients.

## Funding

The authors have nothing to report.

## Ethics Statement

This study was conducted in accordance with the Declaration of Helsinki and approved by the Institutional Ethics Committee of Strasbourg University Hospital (CE‐2025‐90).

## Conflicts of Interest

Laurent SATTLER and Dominique DESPREZ received reimbursement for attending a symposium, as well as speaker and consultancy fees from Sobi. Jordan WIMMER received reimbursement for attending a symposium, as well as speaker fees from Sobi. Léa PIERRE received reimbursement for attending a symposium from Sobi. The remaining authors declare no conflict of interest related to the submitted work.

## Data Availability

The data that support the findings of this study are available from the corresponding author upon reasonable request.
